# Changes in Use of Hepatitis C Direct-Acting Antivirals After Access Restrictions Were Eased by State Medicaid Programs

**DOI:** 10.1001/jamahealthforum.2024.0302

**Published:** 2024-04-05

**Authors:** Sonya Davey, Kevin Costello, Massimiliano Russo, Suzanne Davies, Hussain S. Lalani, Aaron S. Kesselheim, Benjamin N. Rome

**Affiliations:** 1 Program On Regulation, Therapeutics, and Law, Division of Pharmacoepidemiology and Pharmacoeconomics, Department of Medicine, Brigham and Women’s Hospital, Boston, Massachusetts; 2 Center for Health Law and Policy Innovation, Harvard Law School, Cambridge, Massachusetts; 3 The Ohio State University, Department of Statistics, Columbus; 4 Harvard Medical School, Boston, Massachusetts

## Abstract

**Importance:**

Direct-acting antivirals (DAAs) are safe and highly effective for curing hepatitis C virus (HCV) infection, but their high cost led certain state Medicaid programs to impose coverage restrictions. Since 2015, many of these restrictions have been lifted voluntarily in response to advocacy or because of litigation.

**Objective:**

To estimate how the prescribing of DAAs to Medicaid patients changed after states eased access restrictions.

**Design, Setting, and Participants:**

This modified difference-in-differences analysis of 39 state Medicaid programs included Medicaid beneficiaries who were prescribed a DAA from January 1, 2015, to December 31, 2019. DAA coverage restrictions were measured based on a series of cross-sectional assessments performed from 2014 through 2022 by the US National Viral Hepatitis Roundtable and the Center for Health Law and Policy Innovation.

**Exposure:**

Calendar quarter when states eased or eliminated 3 types of DAA coverage restrictions: limiting treatment to patients with severe liver disease, restricting use among patients with active substance use, and requiring prescriptions to be written by or in consultation with specialists. States with none of these restrictions at baseline were excluded.

**Main Outcomes and Measures:**

Quarterly number of HCV DAA treatment courses per 100 000 Medicaid beneficiaries.

**Results:**

Of 39 states, 7 (18%) eliminated coverage restrictions, 25 (64%) eased restrictions, and 7 (18%) maintained the same restrictions from 2015 to 2019. During this period, the average quarterly use of DAAs increased from 669 to 3601 treatment courses per 100 000 Medicaid beneficiaries. After states eased or eliminated restrictions, the use of DAAs increased by 966 (95% CI, 409-1523) treatment courses per 100 000 Medicaid beneficiaries each quarter compared with states that did not ease or eliminate restrictions.

**Conclusions and Relevance:**

The results of this study suggest that there was greater use of DAAs after states relaxed coverage restrictions related to liver disease severity, sobriety, or prescriber specialty. Further reductions or elimination of these rules may improve access to a highly effective public health intervention for patients with HCV.

## Key Points

**Question:**

Did the use of hepatitis C direct-acting antivirals increase after state Medicaid programs eased coverage restrictions associated with liver disease severity, sobriety, or prescriber specialty?

**Findings:**

In this difference-in-differences analysis of 39 Medicaid programs, 32 eased or eliminated restrictions from 2015 to 2019, and these coverage changes were associated with an increase of 966 (95% CI, 409-1523) direct-acting antiviral treatment courses per 100 000 Medicaid beneficiaries per quarter compared with states without changes.

**Meaning:**

The results of this study suggest that Medicaid coverage restrictions were associated with a slowed uptake of a costly but highly effective public health intervention and that further loosening of these restrictions may improve access to curative hepatitis C virus treatment.

## Teaser

This difference-in-differences analysis examines the changes in the prescribing of direct-acting antivirals to Medicaid patients after states eased access restrictions.

## Introduction

More than 2 million individuals in the US have chronic hepatitis C virus (HCV) infection.^1,2^ Complications from HCV were responsible for more than 14 000 deaths in the US in 2019, which was more than the next 60 infectious diseases combined before the COVID-19 pandemic.^3^ Injection drug use, fueled by the ongoing opioid epidemic, was associated with a 400% increase in new HCV infections from 2004 to 2014.^4^

Until recently, treatments for HCV were ineffective and poorly tolerated. However, novel direct-acting antivirals (DAAs) are well tolerated and provide sustained virologic suppression for 95% of patients without cirrhosis.^5^ The first highly efficacious DAA, sofosbuvir, was approved in 2013 and introduced at a price exceeding $80 000 for a 12-week treatment course.^6^ This cost did not include adjuvant therapies, like ribavirin, that were used concurrently with early DAAs. Although sofosbuvir was cost-effective even at such a high price,^7^ the substantial cost of these drugs combined with a high prevalence of disease was associated with an 18% increase in national per capita prescription drug spending between 2013 and 2015,^8^ straining budgets of public and private payers. To control the high upfront cost of treatment, payers imposed varying degrees of restrictions that limited access to DAAs.^9^

These restrictions were imposed by many public and private payers but were particularly notable among state Medicaid programs, which cover an estimated 80% of patients with a diagnosis of HCV.^10^ Medicaid programs are partially funded by states, most of which must operate with a balanced budget; as a result, the cost of DAAs threatened steep cuts in other state-funded programs.^9^ To address these concerns, state Medicaid programs enacted coverage restrictions for this class of drugs. Three of the most common restrictions included limiting treatment to those with severe liver disease, restricting use among those with active substance use, and requiring prescriptions be written by or in consultation with specialists. In 2014, of 42 jurisdictions (including Washington, DC) with publicly available treatment criteria, 34 (81%) restricted coverage based on liver disease severity, 37 (88%) based on drug or alcohol use, and 29 (69%) based on specialist prescriber requirements.^11^

Since 2015, national guidelines promulgated by specialist associations recommended treatment for individuals infected with HCV without considering liver disease severity, sobriety status, or prescriber specialty.^12^ These guidelines were based on evidence that universal treatment was safe, efficacious, and cost-effective.^13,14,15^ In a wave of legal battles against state Medicaid programs, advocates successfully argued that the restrictions violated federal law.^16,17^

From 2015 to 2023, many state Medicaid programs eased or eliminated DAA coverage restrictions.^18^ In some cases, states eased restrictions following policy reform efforts by patients, clinicians, policy advocates, attorneys, and other stakeholders. Other states removed restrictions in response to lower prices offered by manufacturers, in part due to competition among multiple DAAs that were launched during this period. In other cases, restrictions were overturned following lawsuits brought by Medicaid patients.^16,17^ As of 2022, lawsuits had overturned Medicaid DAA coverage restrictions in several states, including Washington, Missouri, Michigan, Colorado, Indiana, Kansas, and Texas.^18^ To better understand the extent to which these restrictions limited treatment of HCV among Medicaid beneficiaries, we performed a difference-in-differences analysis to measure whether the use of DAAs increased in states that lifted or eased restrictions compared with states with no changes.

## Methods

We studied quarterly use of DAAs in 51 Medicaid programs (50 states plus Washington, DC) from January 1, 2015, to December 31, 2019. This period was selected to begin after the first combination DAA (ledipasvir/sofosbuvir) was introduced during the last quarter of 2014, and national hepatology and infectious disease associations recommended treatment of all individuals with chronic HCV with DAAs (except those with limited life expectancy due to nonhepatic causes).^12^ We ended the study before 2020 to avoid confounding by changes in access to treatment that occurred during the COVID-19 pandemic. This study was not submitted for institutional review board approval because it used aggregated, publicly available data that did not qualify as human participants research.

Among 51 Medicaid programs, we excluded 5 with suppressed DAA use data due to a small number of prescriptions (Iowa, North Dakota, Nebraska, South Dakota, and Wyoming) and 5 with missing Medicaid enrollment data (Alaska, Arizona, Washington, DC, Hawaii, and Tennessee). We excluded Massachusetts because it had no DAA restrictions related to liver disease severity, sobriety, or prescriber type throughout the study period. Because Medicaid expansion under the Affordable Act could confound the results, we excluded Maine because it enacted Medicaid expansion in 2019 around the same time DAA restrictions were relaxed, and we censored Virginia starting in the third quarter of 2018 after Medicaid expansion was enacted. We censored 2 states (Louisiana and Washington) starting in the third quarter of 2019 after they reportedly switched some of their Medicaid population to a subscription-based payment system for DAAs.^19^

### Defining Changes in Medicaid Restrictions

For each state Medicaid program, we measured DAA coverage restrictions based on a series of cross-sectional assessments performed from 2014 through 2022 by the National Viral Hepatitis Roundtable and the Center for Health Law and Policy Innovation.^11,18^ These initial assessments were based on published Medicaid documents and direct communications with state Medicaid offices. For this analysis, 2 authors (K.C. and S.D.) reviewed additional documents, including archived Medicaid documents and secondary sources when necessary to identify dates when relevant policies were changed, including for quarters not covered by the original assessments.

Coverage restrictions were measured in 3 domains: requirements for minimum disease severity measured with the liver fibrosis score, requirements for periods of sobriety before treatment, and limited prescribing to certain specialists. For each domain, we stratified each state’s policies as either strict, lenient, or no restriction, similar to a prior study (Table 1^20^).^21^ For disease restrictions, limiting treatment to those with fibrosis scores of F3 or F4 was considered strict and fibrosis scores of F1 or F2 was considered lenient; for prescriber restrictions, limiting prescriptions to specialist prescribers was considered strict and limiting prescriptions to clinicians with specialist consultation was considered lenient; for sobriety restrictions, any length of required sobriety period was considered strict and mandatory sobriety counseling considered lenient.

**Table 1.** Medicaid Prescribing Restrictions for Hepatitis C Virus Direct-Acting Antivirals

Restriction type	Strict	Lenient	None
Liver disease severity^a^	Fibrosis score ≥F3	Fibrosis score of F1 or F2	No restriction
Prescriber	Must be prescribed by a specialist^b^	Must be prescribed in consultation with a specialist^b^	No restriction
Sobriety	Any length of sobriety restriction	Prescriber must counsel patient about sobriety	No restriction

^a^ The fibrosis score is categorized as F0 through F4, with F0 indicating no liver fibrosis, F1 indicating mild, F2 indicating moderate, F3 indicating severe liver fibrosis, and F4 demonstrating cirrhosis. Liver biopsy is considered the criterion standard for assessing liver fibrosis; however, other methods to evaluate fibrosis include laboratory testing, liver ultrasonography, and elastography.^20^

^b^ Specialists are physicians trained in infectious diseases and gastroenterology.

For each state, we identified calendar quarters during which there was a change in any of the 3 restriction domains (eTable 1 in Supplement 1). For states with multiple sequential changes, in our primary analysis, we selected the first change during the study period. In a sensitivity analysis, we included the first major change; major changes were defined as simultaneously easing restrictions in 2 or more domains or changing any domain from strict to no restriction (eTable 2 in Supplement 1).

### Quarterly Use of DAAs

The primary outcome was the number of DAA treatment courses per 100 000 adult Medicaid enrollees in each state. This was calculated based on the quarterly number of units (ie, tablets) of each DAA reimbursed by fee-for-service or managed care Medicaid plans using public Medicaid State Drug Utilization data.^22^ For each DAA, we converted the number of units to the number of HCV treatment courses based on the recommended dosing from the US Food and Drug Administration (FDA) labeling (eTable 3 in Supplement 1). State data for specific drugs were suppressed if fewer than 11 units were reimbursed in a given quarter due to federal privacy protection rules.^22^ To address these missing data, we excluded states if more than two-thirds of the quarterly DAA data were suppressed. We excluded use of daclatasvir and simeprevir to prevent double counting, because the FDA has only approved these drugs for combination use with another DAA.

To compare DAA use between states, we calculated the number of treatment courses per 100 000 adult Medicaid enrollees using public Medicaid enrollment files.^23^ We excluded states in which quarterly enrollment data were unavailable and excluded specific quarters for which enrollment was not available in California (quarter 1 in 2015 to quarter 1 in 2016) and New Mexico (quarter 1 in 2015 to quarter 2 in 2016).

### State Characteristics

We compared baseline characteristics of states that eased restrictions from 2015-2019 vs those that did not ease restrictions during this time. These included baseline DAA restrictions in 2015 (disease severity, sobriety, and prescriber specialty), whether the state had enacted Medicaid expansion under the Affordable Care Act as of January 1, 2016,^24^ and US Census region.

We stratified states based on whether DAAs were reimbursed predominantly (>90%) by fee-for-service Medicaid, predominantly (>90%) by managed care organizations, or through a combination of fee-for-service and managed care Medicaid. Under federal law, managed care organizations cannot impose more restrictive DAA coverage standards than fee-for-service plans,^25^ and some state reports to US Centers for Medicare & Medicaid Services memorialize efforts to impose such minimum pharmaceutical coverage requirements.^22^ But enforcement of this parity requirement is limited, and in practice, some managed care organizations impose stricter criteria than the state requirements.^18^ We also stratified states into tertiles as low, medium, or high HCV prevalence based on 2013 to 2016 data from the National Health and Nutritional Examination Survey.^26^

### Statistical Analysis

We compared baseline characteristics of states with any vs no change in restrictions using χ^2^ tests. We assessed trends in mean state quarterly DAA use among states before vs after easing or eliminating coverage restrictions. To prevent the results from being skewed toward more populous states, each state was weighted equally.

We performed a difference-in-differences analysis to determine whether the use of DAAs increased after states eased prescribing restrictions compared with states that did not ease restrictions. Traditional difference-in-difference models estimate the pre-post change for 2 groups around a single point, but in this study, states enacted changes at multiple points between 2015 and 2019. To address this, we used a modified difference-in-differences model described by Callaway and Sant’Anna^27^ that estimates an average treatment effect for multiple difference-in-differences models, grouping states based on the quarter in which DAA restrictions were eased. In each model, states that did not ease restrictions at the point of interest contributed to the unexposed group until they eased restrictions or were censored. Unlike standard difference-in-differences models, this modified approach was robust to treatment effect heterogeneity and dynamics and has been shown to provide sensible results in practice.^27^

In the combined difference-in-differences model, the average treatment effect represented the change in the number of HCV treatment courses per 100 000 Medicaid beneficiaries per quarter after states eased or eliminated coverage restrictions compared with states that did not ease restrictions. In addition to the overall treatment effect, we calculated the average treatment effect in the 5 quarters before and after restrictions were eased, which we used to verify that the parallel trends assumption was satisfied.

We performed several other sensitivity analyses. First, we included the first major change in each state, as described previously. Second, we restricted the analysis period to 4 quarters before and after states eased or eliminated restrictions, because points further from the change may have been affected by other temporal trends or events. Third, we stratified our analysis by states in which DAAs were reimbursed predominantly (>90%) by fee-for-service or managed care Medicaid. For this analysis, we excluded 5 states with fee-for-service and managed care reimbursement of DAAs and 3 states that carved DAAs out of managed care coverage during the study period.^28^ Michigan also carved out DAAs from its managed care plans in 2015, but this occurred 3 years before DAA coverage restrictions were relaxed in 2018, so we included Michigan as a predominantly fee-for-service state. Fourth, states were stratified as low, medium, or high HCV prevalence based on tertiles.^26^ Fifth, we stratified by states that eased restrictions through 2017 quarter 2 vs 2017 quarter 3 or later, because around this time, newer DAAs were introduced that treated more HCV genotypes, and net costs for DAAs began to decrease.^29^ Sixth, we separately analyzed the average treatment effect for states that changed restrictions in each of the 3 domains (disease severity, sobriety, and prescriber) to determine if 1 type of coverage restriction particularly limited patient access to DAAs. States that changed multiple domains simultaneously were included in each domain. Analyses were conducted in R, version 4.2.2 (R Foundation).

## Results

### DAA Coverage Restrictions

Among the 39 Medicaid programs included in the study, 7 states (18%) eliminated all 3 DAA coverage restrictions, 25 (64%) eased restrictions, and 7 (18%) maintained the same restrictions during the study period (eTable 1 in Supplement 1). One state (South Carolina) initially eased restrictions but subsequently added a strict sobriety restriction during the first quarter of 2019, after which data were censored. Among the 32 states that eased or eliminated restrictions, 23 (72%) had at least 1 major change, and 9 states (28%) had only minor changes; 3 states (9%) had a minor change followed by a major change (eTable 2 in Supplement 1).

Compared with the states with no changes, those that eased or eliminated restrictions were more likely to have strict baseline restrictions for disease severity (75% vs 43%) (Table 2^26^). Otherwise, there was no significant differences between states that eased restrictions vs states that did not ease restrictions in terms of baseline prescriber or sobriety restrictions, source of Medicaid reimbursement (predominantly fee-for-service vs managed care vs both), state HCV prevalence, Medicaid expansion status, and US region (Table 2).

**Table 2.** Characteristics of States That Eased vs Did Not Ease DAA Coverage Restrictions

Characteristic	Did not ease restrictions (n = 7), No. (%)	Eased restrictions (n = 32), No. (%)	*P* value^a^
Liver disease restriction in 2015^b^			
None	3 (42.9)	0	<.001
Lenient	1 (14.3)	8 (25.0)	
Strict	3 (42.9)	24 (75.0)	
Prescriber restriction in 2015^b^			
None	2 (28.6)	6 (18.8)	.78
Lenient	3 (42.9)	13 (40.6)	
Strict	2 (28.6%	13 (40.6)	
Sobriety restriction in 2015^b^			
None	0	4 (12.5)	.60
Lenient	1 (14.3)	5 (15.)	
Strict	6 (85.7)	23 (71.9)	
Source of Medicaid DAA reimbursement^c^			
Predominantly (>90%) fee for service	3 (42.9)	11 (34.4)	.66
Predominantly (>90%) managed care organizations	2 (28.6)	15 (46.9)	
Both fee-for-service and managed care organizations	2 (28.6)	6 (18.8)	
State HCV prevalence^d^			
Low	3 (42.9)	10 (31.3)	.84
Medium	2 (28.6)	11 (34.4)	
High	2 (28.6)	11 (34.4)	
Medicaid expanded status^e^			
No	4 (57.1)	10 (31.3)	.39
Yes	3 (42.9)	22 (68.8)	
US Census region			
Midwest	1 (14.3)	7 (21.9)	.22
South	5 (71.4)	10 (31.3)	
West	1 (14.3)	8 (25.0)	
Northeast	0	7 (21.9)	

Abbreviations: DAA, direct-acting antiviral; HCV, hepatitis C virus.

^a^ From χ^2^ tests.

^b^ Restriction categories are described in Table 1.

^c^ States were categorized based on the proportion of DAA units reimbursed via fee-for-service or managed care divided by the total number of DAA units reimbursed during the study period.

^d^ States were divided into tertiles based on the prevalence of HCV from 2013 to 2016 from Rosenberg et al.^26^

^e^ Denotes whether states had expanded Medicaid under the Affordable Care Act as of January 1, 2016.

### Use of DAAs

**Figure 1.**
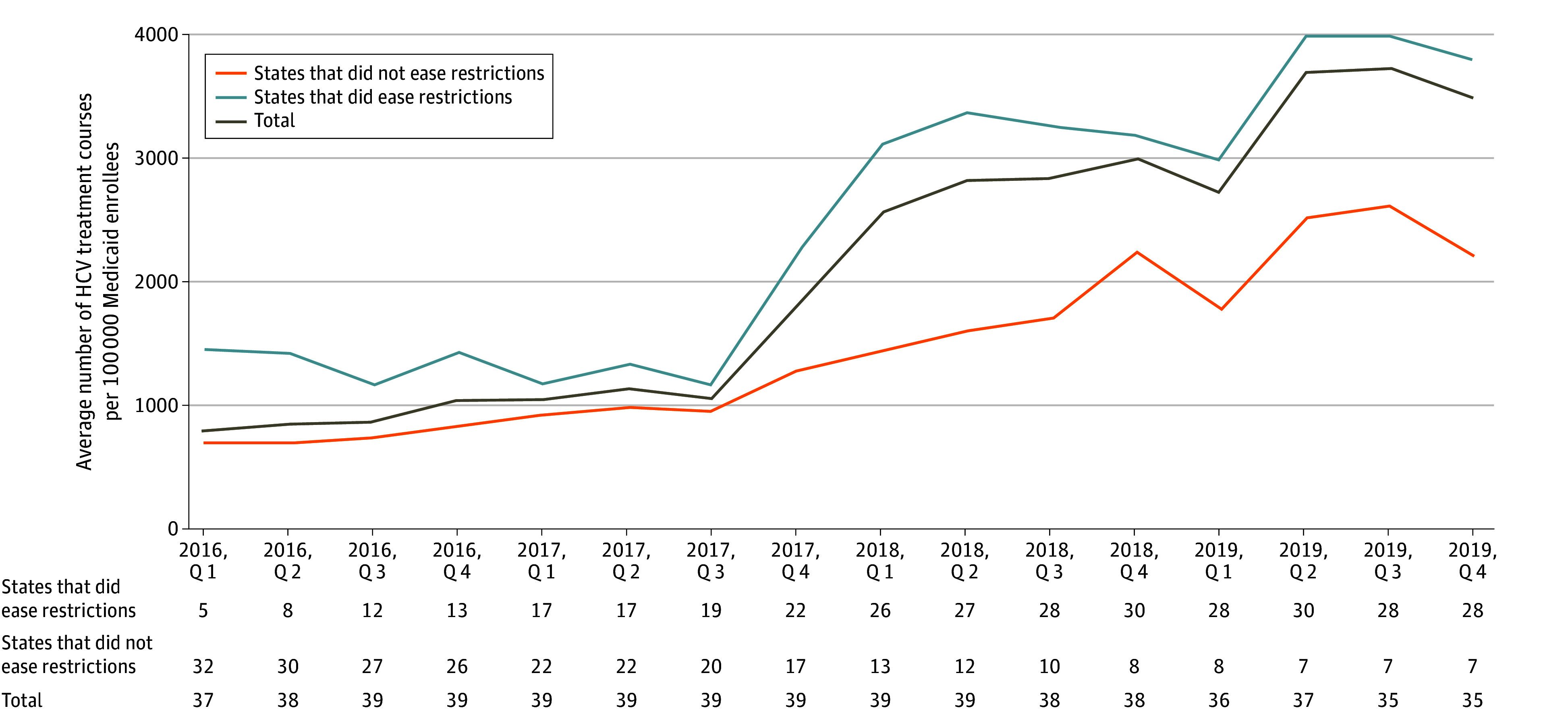
Trends Over Time in Use of Direct-Acting Antivirals Among States That Did Not Ease Restrictions vs Those That Did The brown line shows the mean number of hepatitis C virus (HCV) treatment courses per 100 000 Medicaid enrollees from 2016 to 2019. The blue and orange lines stratify states based on whether they had eased or eliminated coverage restrictions up to that point. States changed from the did not ease restrictions to the did ease restrictions categories in the quarter (Q) when a change was made. The number of states in each group is shown below the x-axis. The average number of HCV treatment courses is an unweighted average of the states.

The mean use of HCV treatment in all states increased from 669 treatment courses per 100 000 Medicaid beneficiaries in the first quarter of 2015 to 3601 per 100 000 in the last quarter of 2019 (Figure 1). Use of DAAs increased even among states that did not ease restrictions; states that had previously eased restrictions had higher DAA use than those that had not yet eased restrictions in each quarter from 2015 to 2019.

### Primary Analysis

**Figure 2.**
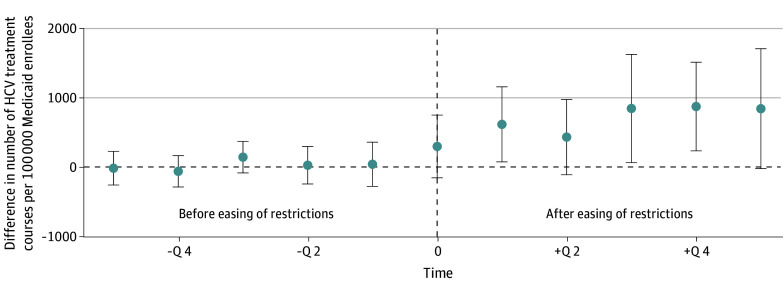
Average Difference in Direct-Acting Antiviral (DAA) Use in States That Eased vs Did Not Ease Coverage Restrictions Each point shows the average difference in the number of DAA treatment courses per 100 000 Medicaid beneficiaries between states that eased restrictions and those that did not. Values greater than 0 represent higher use of DAAs in states that eased restrictions compared with those that did not. Time 0 is the calendar quarter (Q) during which the restrictions were eased, and the effect estimates in the 5 quarters before vs after this change are averaged across models for states that eased restrictions at different times from 2015 to 2019. Whiskers represent 95% CIs. HCV indicates hepatitis C virus.

In the primary difference-in-differences model, easing of restrictions was associated with an average increase of 966 (95% CI, 395-1537) DAA treatment courses per 100 000 Medicaid beneficiaries each quarter compared with states that did not ease restrictions. Before states eased restrictions, there was no difference in DAA use between states that eased vs did not ease restrictions; after states eased restrictions, the increase in DAA use was abrupt and sustained for at least 5 quarters (Figure 2).

### Sensitivity Analyses

**Figure 3.**
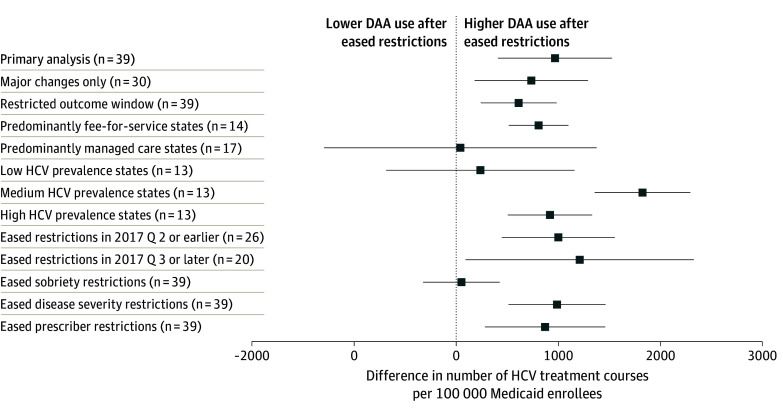
Average Difference-in-Differences Estimates for Sensitivity Analyses Each row represents the average difference in the number of direct-acting antiviral (DAA) treatment courses per 100 000 Medicaid enrollees per quarter (Q) in states that eased coverage restrictions compared with those that did not. Positive differences represent higher DAA use in states that eased restrictions. Results are shown for the primary analysis and 12 sensitivity analyses. Whiskers represent 95% CIs. HCV indicates hepatitis C virus.

The results of sensitivity analyses are shown in Figure 3. Results were similar to the primary analysis when limiting to states with at least 1 major change in DAA restrictions (735; 95% CI, 182-1288) or restricting the outcome window to only 4 quarters before and after easing of restrictions (611; 95% CI, 241-982). Results were also similar to the primary analysis when limiting the analysis to predominantly fee-for-service Medicaid states (806; 95% CI, 515-1097). When limiting to states in which DAAs were predominantly reimbursed by managed care organizations, there was no significant change in DAA use after states eased restrictions (43; 95% CI, −1287 to 1372). There was also no increase in DAA use after restrictions were eased in states with low HCV rates (238; 95% CI, −681 to 1157), but there was an increase in states with medium (1820; 95% CI, 1353-2287) and high HCV prevalence (917; 95% CI, 506-1329). There was a significant increase in DAA use whether states eased restrictions from 2016 through 2017 quarter 2 (999; 95% CI, 448-1550) or in 2017 quarter 3 through 2019 (1207; 95% CI, 93-2321).

When examining each of the 3 domains separately, easing or eliminating restrictions related to liver disease severity (986; 95% CI, 512-1460) and prescriber specialty (869; 95% CI, 283-1456) were associated with increased use of DAAs, while changes in sobriety restrictions were not associated with a change in DAA use (53; 95% CI, −321 to 427). The parallel trend assumption was met in each sensitivity analysis (eFigure 2 in Supplement 1).

## Discussion

In this difference-in-differences analysis, we found that 32 state Medicaid programs that eased or eliminated coverage restrictions for DAAs treated significantly more Medicaid beneficiaries for HCV compared with states that maintained restrictions. These results provide evidence that Medicaid policies that restricted access to patients based on liver disease severity, sobriety, or prescriber specialty were associated with a reduced number of patients treated for HCV from 2015 to 2019. While many states eased restrictions during the study period, 22 states continued to impose 1 or more of these restrictions as of 2022^18^; removing these restrictions may further expand access to HCV treatment.

These study results align with other more limited studies that estimated whether eliminating Medicaid HCV treatment restrictions was associated with increased DAA use. Two cross-sectional studies observed higher use of DAAs in states with fewer Medicaid HCV treatment restrictions.^21,30^ Other studies have identified increases in HCV treatment rates after changes to Medicaid coverage restrictions in Indiana,^31^ 4 New England states,^32^ and Oregon.^33^

To our knowledge, this study is the first to comprehensively evaluate the effect of easing DAA Medicaid coverage restrictions across the US. This approach had several strengths. First, by averaging the treatment effect across 39 states, our study had increased power and precision. Second, our results are more generalizable than previous studies, demonstrating a robust effect across states with varying demographic and sociopolitical contexts.

A notable finding from this study was that changes in easing of state restrictions were associated with higher DAA use when DAAs were reimbursed predominantly via fee-for-service Medicaid but not managed care organizations. The most likely explanation is that DAA coverage policies in managed care organizations sometimes deviate from the statewide policies.^18^ A previous study found that HCV DAA use increased after 4 states carved out coverage of DAAs from Medicaid managed care organizations.^28^ Together, these findings suggest that better state and federal enforcement is needed to ensure that managed care organizations offer DAA coverage that is no more restrictive than the state rules.

We also found that DAA use did not increase significantly after states eased sobriety restrictions, which was different from the overall findings and results of the 2 other restriction categories. This may be because clinicians continued to have stigma about treating HCV in patients with active substance use regardless of whether such treatment was reimbursable. There is evidence that treatment with DAAs is effective at preventing long-term complications of HCV among those with active substance use.^34^ Our findings suggest that more education is needed to expand treatment access to this population of patients.

From 2015 to 2019, there was an increase in the number of Medicaid patients treated with DAAs even among states that did not ease restrictions. There are several likely reasons for this increased use of this new class of drugs, including more familiarity by patients and prescribers, expanded use of the drugs to treat additional HCV genotypes, and decreases in the cost of DAAs. Costs declined in part due to competition between AbbVie and Gilead; in 2017, both firms introduced authorized generic DAAs priced at $36 000 and $24 000 per course of treatment, respectively, which was much lower than the original list price for DAAs.^29^ Lower prices may have contributed to some states’ willingness to ease DAA coverage restrictions.

Coverage restrictions are one of many barriers to widespread treatment of HCV. The US Centers for Disease Control and Prevention estimates that 40% of those with HCV are unaware that they are infected.^35^ In 2020, the US Preventive Services Task Force recommended universal HCV screening for all adolescent and adult patients improve detection of asymptomatic chronic disease that could be treated before complications arise.^36^ Once patients receive a diagnosis, many do not receive timely treatment. Among Medicaid enrollees with a new diagnosis of HCV from January 2019 to October 2020, only 23% received treatment within 1 year after diagnosis.^10^ More than half of these Medicaid enrollees with HCV lived in states with treatment restrictions, and these enrollees were 23% less likely to receive treatment within 1 year after diagnosis compared with those living in states without restrictions.^10^ However, other factors that limit timely treatment include preauthorization requirements, fragmented care of those at highest risk for HCV, and limited overall capacity for screening and treatment.

These results have policy implications. Although 32 states eased or eliminated coverage restrictions during the past decade, many states continue to impose some restrictions. As of June 2023, 1 state had liver disease severity restrictions, 10 states had sobriety restrictions, and 4 states had prescriber restrictions.^18^ Our results suggest that easing or eliminating these remaining restrictions may increase access to HCV treatment in these states.

In March 2023, the White House and National Institutes of Health announced a National Program for Hepatitis C Elimination, with the goal of addressing HCV through a multimodal approach of improved HCV outreaching, screening, and treatment.^20^ Our results suggest that the remaining Medicaid coverage restrictions will be a barrier to achieving this goal.

### Limitations

Our study had several limitations. We focused on 3 common Medicaid coverage restrictions but we did not account for changes in other restrictions, including prior authorization requirements, required documentation of HCV genotype, prohibitions on replacement of lost or stolen medications, review of past adherence to other prescribed medications, documentation of psychiatric and/or housing stability, and requirements to fill DAAs at specialty pharmacies.^18^ Our results among Medicaid recipients may not be generalizable to those with private insurance or Medicare part D plans, which also imposed restrictions to DAAs during this time. Finally, we calculated DAA treatment courses based on the FDA-recommended duration for treatment-naive patients without decompensated cirrhosis; if some patients deviated from this assumption, the number of patients treated may differ from the number of estimated treatment courses.

## Conclusions

In this study, from 2015 to 2019, treatment of HCV with DAAs increased after state Medicaid programs eased coverage restrictions compared with states that did not ease restrictions. Further reductions or eliminations of these restrictions may maximize the public health effect of these safe and effective treatments for HCV.

## Notes

Supplement 1. eTable 1. List of changes in state Medicaid restrictions for hepatitis C direct-acting antivirals eTable 2. Summary of all states with the type of restriction changes (Major vs Minor) and the number of quarters in which restrictions changes occurred eTable 3. DAAs included in the study eFigure 1. Trends in use of DAAs among states that did not ease restrictions vs states that eased restrictions, with 95% confidence intervals eFigure 2. Sensitivity analyses

Supplement 2. Data sharing statement
