# Supplementary material for: Changes in Use of Hepatitis C Direct-Acting Antivirals After Access Restrictions Were Eased by State Medicaid Programs
Source: JAMA Health Forum. 2024 Apr 5;5(4):e240302. doi: 10.1001/jamahealthforum.2024.0302 (PMC10998155; doi:10.1001/jamahealthforum.2024.0302)
Supplement: Supplement 1. — eTable 1. List of changes in state Medicaid restrictions for hepatitis C direct-acting antivirals eTable 2. Summary of all states with the type of restriction changes (Major vs Minor) and the number of quarters in which restrictions changes occurred eTable 3. DAAs included in the study eFigure 1. Trends in use of DAAs among states that did not ease restrictions vs states that eased restrictions, with 95% confidence intervals eFigure 2. Sensitivity analyses [file jamahealthforum-e240302-s001.pdf]

Supplemental Online Content

Davey S, Costello K, Russo M, et al. Changes in use of hepatitis C direct-acting antivirals after ease of access restrictions in state Medicaid programs. *JAMA Health Forum*. 2024;5(4):e240302. doi:10.1001/jamahealthforum.2024.0302

eTable 1. List of changes in state Medicaid restrictions for hepatitis C direct-acting antivirals
eTable 2. Summary of all states with the type of restriction changes (Major vs Minor) and the number of quarters in which restrictions changes occurred
eTable 3. DAAs included in the study
eFigure 1. Trends in use of DAAs among states that did not ease restrictions vs states that eased restrictions, with 95% confidence intervals
eFigure 2. Sensitivity analyses

This supplemental material has been provided by the authors to give readers additional information about their work.

© 2024 Davey S et al. *JAMA Health Forum.*

eTable 1. List of changes in state Medicaid restrictions for hepatitis C direct-acting antivirals

State	Major vs Minor Change*	Disease	Sobriety	Prescriber	Quarter of Change
AL[0]	-	Lenient	Strict	None	None
AK[1]	-				
AZ[1]	-				
AR[0]	-	Strict	Strict	Strict	None
CA	*Censored[1a]*	Lenient	Strict -> None	Lenient -> None	2015Q3
CA	Minor[8]	Lenient -> None	None	None	2018Q3
CO	Major[8,9]	Strict -> Lenient	Strict -> Lenient	Lenient	2016Q4
CO	Minor	Lenient -> None	Lenient	Lenient	2018Q1
CT	Major[8,9]	Strict -> None	Strict -> None	Lenient -> None	2015Q3
DC[1]	-				
DE	Major[8,9]	Strict -> Lenient	Strict -> Lenient	None	2017Q1
DE	Minor	Lenient -> None	Lenient	None	2018Q1
FL	Major[8,9]	Strict -> None	Strict	Lenient	2016Q2
GA[0]	-	None	Lenient	None	None
HI[1]	-				
IA[2]	-				
ID	Minor[8]	Strict -> Lenient	Strict	Lenient	2016Q3
ID	Minor	Lenient -> None	Strict	Lenient	2019Q3
IL	Major[8,9]	Strict -> None	Strict -> Lenient	Lenient	2018Q4
IN	Minor[8]	Strict	None	Strict -> Lenient	2015Q3
IN	Minor	Strict -> Lenient	None	Lenient	2016Q3
IN	Minor	Lenient -> None	None	Lenient	2019Q3
KS	Major[8,9]	Strict-> None	Strict	Lenient -> None	2019Q2
KY	Major[8,9]	Lenient -> None	Strict -> Lenient	Strict	2017Q4
LA	Major[8,9]	Strict	Strict -> Lenient	Strict -> None	2018Q2
LA	*Censored[4]*	Strict -> None	Lenient -> None	None	2019Q3
MA[3]	-				
MD	Major[8,9]	Lenient	Strict -> Lenient	Strict -> Lenient	2017Q3
ME[6]	-	-	-	-	-
MI	Minor[8]	Strict -> Lenient	Strict	Lenient	2018Q4
MN[0]	-	None	Strict	Strict	None
MO	Minor[8]	Strict	Strict -> Lenient	None	2017Q1
MO	Major[9]	Strict -> None	Lenient	None	2017Q4

© 2024 Davey S et al. *JAMA Health Forum.*

State	Major vs Minor Change*	Disease	Sobriety	Prescriber	Quarter of Change
MS[0]	-	None	Strict	Lenient	None
MT[0]	-	Strict	Strict	Lenient	None
NC	Minor[8]	Lenient -> None	Strict	None	2017Q4
ND[2]	-				
NE[2]	-				
NH	Major[8,9]	Strict -> None	Strict	Strict -> Lenient	2016Q3
NJ	Major[8,9]	Strict -> Lenient	Lenient -> None	Strict	2016Q2
NJ	Minor	Lenient -> None	None	Strict	2018Q3
NM	Minor[8]	Lenient -> None	Lenient	None	2017Q4
NV	Major[8,9]	Strict -> None	None	None	2016Q1
NY	Major[8,9]	Strict -> None	Lenient	Strict -> Lenient	2016Q1
NY	Minor	None	Lenient	Lenient -> None	2018Q3
OH	Minor[8]	Strict -> Lenient	Strict	Strict	2017Q3
OH	Major[9]	Lenient -> None	Strict	Strict -> Lenient	2019Q1
OK	Major[8,9]	Lenient -> None	Strict -> Lenient	Lenient	2018Q1
OR	Major[8,9]	Strict -> Lenient	Strict	Lenient -> None	2018Q1
PA	Major[8,9]	Lenient -> None	Lenient	Strict -> Lenient	2018Q1
PA	Minor	None	Lenient	Lenient -> None	2018Q4
RI	Major[8,9]	Strict -> None	Strict -> None	Strict -> None	2018Q3
SC	Major[8,9]	Strict-> Lenient	Strict -> Lenient	Strict-> Lenient	2016Q3
SC	Minor	Lenient -> None	Lenient	Lenient	2017Q1
SC	*Censored*[5]	None	Lenient -> Strict	Lenient	2019Q1
SD[2]	-				
TN[1]	-				
TX[0]	-	Strict	Strict	Lenient	None
UT	Minor[8]	Lenient -> None	None	Lenient	2018Q1
VA	Minor[8]	Strict -> Lenient	Lenient	Lenient	2016Q3
VA	Minor	Lenient -> None	Lenient	Lenient	2017Q1
VA	*Censored*[7]	None	Lenient -> None	Lenient -> None	2018Q3
VT	Major[8,9]	Strict -> Lenient	Strict -> None	Lenient	2017Q1
VT	Minor	Lenient -> None	None	Lenient	2018Q1
WA	Major[8,9]	Strict -> None	Strict -> None	Strict	2016Q2

© 2024 Davey S et al. *JAMA Health Forum.*

State	Major vs Minor Change*	Disease	Sobriety	Prescriber	Quarter of Change
WA[4]	*Censored[4]*	None	None	Strict -> None	2019Q3
WI	Minor[8]	Strict	Strict	Strict -> Lenient	2015Q2
WI	Minor	Strict -> Lenient	Strict	Lenient	2016Q3
WI	Major[9]	Lenient -> None	Strict	Lenient -> None	2017Q3
WI	Major	None	Strict -> None	None	2019Q3
WV	Minor[8]	Strict -> Lenient	Strict	Lenient	2017Q1
WY[2]	-				

* Major change is defined as 1 or more changes from Strict -> None or 2 or more changes in one quarter. Minor change is defined as 1 change from Lenient -> None.

0. State had no changes in restrictions during the study period

States that were excluded from the study are shaded in gray with the reason for state exclusion detailed in the table footnote. There are four restriction changes that were censored with the reason for restriction change censoring detailed in the table footnotes. For states that eased restrictions in the study, each change that occurred between 2015 and 2019 is listed and categorized as a major vs minor change. Changes included in the primary and/or sensitivity analysis are noted.

Reason for Exclusion or Censoring:

1. Excluded because the number of adult Medicaid enrollees was not available from public sources.

1a. Censored change because for California, the number of adult Medicaid enrollees was not available until 2016Q2.

2. Excluded because more than two-thirds of the HCV DAA use data was suppressed during the study period due to <11 users.
3. State with no restrictions throughout the study period
4. Censored change because changes in LA (2019Q3) and Washington (2019Q4) occurred simultaneous with implementation of a subscription payment model.
5. Censored change because the change in SC was the only case in which coverage became more restrictive in 2019Q1.
6. Excluded because Medicaid Expansion occurred during the study period and concurrently with easing of restriction.
7. Censored change Medicaid Expansion occurred 2019 Q1.

Primary and Sensitivity Analysis:

8. Included in the primary analysis
9. Included in the sensitivity analysis of first change major

© 2024 Davey S et al. *JAMA Health Forum.*

eTable 2. Summary of all states with the type of restriction changes (Major vs Minor) and the number of quarters in which restrictions changes occurred.

**Types of Restriction Changes**	**States**
1 Major Change [13 states]	CT, FL, IL, KS, KY, LA, MD, NH, NV, OK, OR, RI, WA
1 Major Change followed by 1 Minor Change [7 states]	CO, DE, NJ, NY, PA, SC, VT
1 Minor Change [6 states]	CA, MI, NC, NM, UT, WV
2 Minor Changes [2 states]	ID, VA
3 Minor Changes [1 state]	IN
1 Minor Change Followed by 1 Major Change [2 states]	MO, OH
2 Minor Changes Followed by 1 Major Change Followed by 1 additional Major Change [1 states]	WI

For a sensitivity analysis, the quarter of the first major change was analyzed (23 states with at least one major change). Three states (MO, OH, VA, WI) had a minor change preceding the major change; this was ignored in the sensitivity analysis but the first minor change was included in the primary analysis. There were 9 states with only minor changes; the first minor change was used in the primary analysis.

© 2024 Davey S et al. *JAMA Health Forum.*

eTable 3. DAAs included in the study

**Drug name (Brand Name, Manufacturer)**	**National drug code for adult drug formulation**	**Standard FDA-recommended adult dose**	**Standard FDA-recommended duration**	**Tablets per treatment course**	**Date of approval**
Sofosbuvir (Sovaldi, Gilead)	61958-1501-1	sofosbuvir 400mg	One pill daily for 12 weeks[1]	84 tablets	December 2013
Ledipasvir-sofosbuvir (Harvoni, Gilead)	61958-1801-1	sofosbuvir 400mg/ ledipasvir 90mg	One pill daily for 12 weeks[2]	84 tablets	October 2014
Elbasvir-grazoprevir (Zepatier, Merck)[3]	0006-3074-02	elbasvir 50mg/ grazoprevir 100mg	One pill daily for 12 weeks[4]	84 tablets	January 2016
Sofosbuvir-velpatasvir (Epclusa, Gilead)[5]	61958-2201-1	sofosbuvir 400mg/ velpatasvir 100mg	One pill daily for 12 weeks	84 tablets	June 2016
Sofosbuvi-velpatasvir-voxilaprevir (Vosevi, Gilead)	61958-2401-1	sofosbuvir 400mg/ velpatasvir 100mg/ voxilaprevir 100mg	One pill daily for 12 weeks[6]	84 tablets	July 2017
Glecaprevir-pibrentasvir (Mavyret, AbbVie)	0074-2625-01, 0074-2625-28, 0074-2625-56, 0074-2625-84	glecaprevir 100mg/ pibrentasvir 40mg	Three pills daily for 8 weeks[7]	168 tablets	August 2017
Sofosbuvir-velpatasvir (Epclusa, Asegua)[5]	72626-2701-1	sofosbuvir 400mg/ velpatasvir 100mg	One pill daily for 12 weeks	84 tablets	January 2019
Ledipasvir-sofosbuvir (Harvoni, Asegua)	72626-2601-1	sofosbuvir 400mg/ ledipasvir 90mg	One pill daily for 12 weeks[2]	84 tablets	January 2019

[1] Duration in patients with Genotype 1, 2, or 4 (for Genotype 3, recommended duration is 24 weeks)
[2] Duration in treatment-naïve patients with or without cirrhosis, or treatment-experienced patients without cirrhosis (for treatment-experience patients with cirrhosis recommended duration is 24 weeks)
[3] Should be co-administered with ribavirin except in Genotype 1a patients without baseline NS5A polymorphisms
[4] Duration in Genotype 1a treatment-naïve and treatment-experienced patients without baseline NS5A polymorphisms, Genotype 1b treatment-naïve and treatment-experienced patients, and Genotype 4 treatment-naïve patients (for Genotype 1a treatment-naïve and treatment-experienced patients with baseline NS5A polymorphisms and Genotype 4 treatment-experienced patients, recommended duration is 16 weeks).
[5] Should be co-administered with ribavirin in decompensated cirrhosis
[6] Duration in patients with without cirrhosis or compensated cirrhosis
[7] Duration for treatment-naïve patients without cirrhosis (for treatment-naïve patient with compensated cirrhosis recommended duration is 12 weeks, in treatment-experienced patients with no cirrhosis recommended duration ranges from 8 to 12 weeks depending on previous treatment, and in treatment-experience patients with compensated cirrhosis recommended duration ranges from 12 to 16 weeks depending on previous treatment).

© 2024 Davey S et al. *JAMA Health Forum.*

eFigure 1. Trends in use of DAAs among states that did not ease restrictions vs states that eased restrictions, with 95% confidence intervals

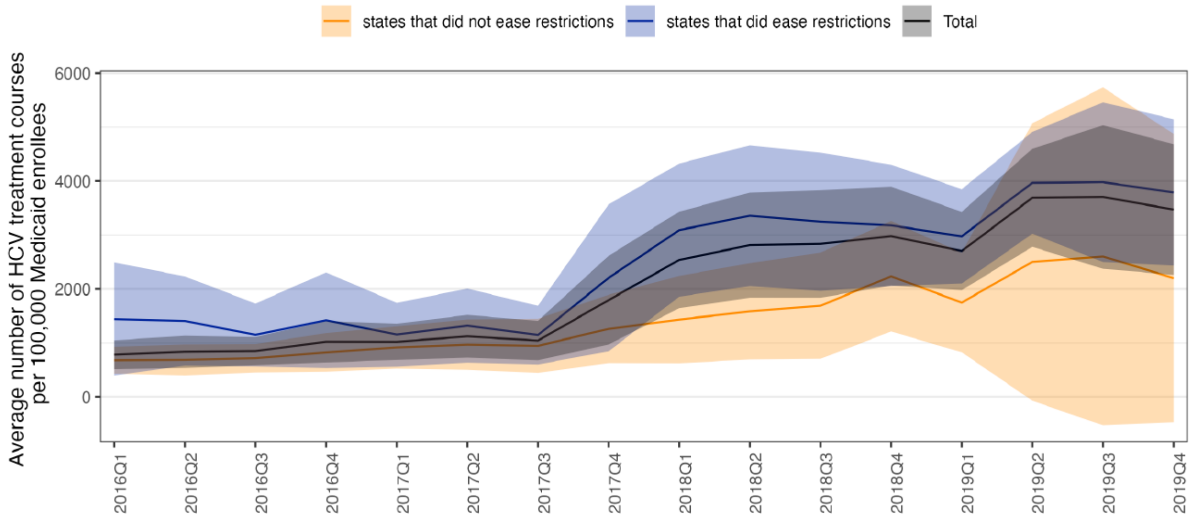

	2016Q1	2016Q2	2016Q3	2016Q4	2017Q1	2017Q2	2017Q3	2017Q4	2018Q1	2018Q2	2018Q3	2018Q4	2019Q1	2019Q2	2019Q3	2019Q4
states that did ease restrictions	5	8	12	13	17	17	19	22	26	27	28	30	28	30	28	28
states that did not ease restrictions	32	30	27	26	22	22	20	17	13	12	10	8	8	7	7	7
Total	37	38	39	39	39	39	39	39	39	39	38	38	36	37	35	35

The black line shows the mean number of HCV treatment courses per 1000,000 Medicaid enrollees from 2016-2019. The blue and orange lines stratify states based on whether they had eased or eliminated coverage restrictions up to that point. States changed from the “did not ease restrictions” to the “did ease restrictions” categories in the quarter when a change was made. The number of states in each group is shown below the x-axis. The average number of HCV treatment courses is an unweighted average of the states. Shaded areas represent 95% confidence intervals.

© 2024 Davey S et al. *JAMA Health Forum.*

eFigure 2:
eFigure 2A to 2L illustrate sensitivity analyses. Each point shows the average difference in the number of DAA treatment courses per 100,000 Medicaid beneficiaries between states that eased restrictions and those that did not ease restrictions. Values greater than 0 represent higher use of DAAs in states that eased restrictions, compared to those that did not ease restrictions. Time 0 is the calendar quarter during which the restrictions were eased, and the effect estimates in the 5 quarters before vs. after this change are averaged across models for states that eased restrictions at different times from 2015-2019. Whiskers represent 95% confidence intervals.

eFigure 2A. Major changes only

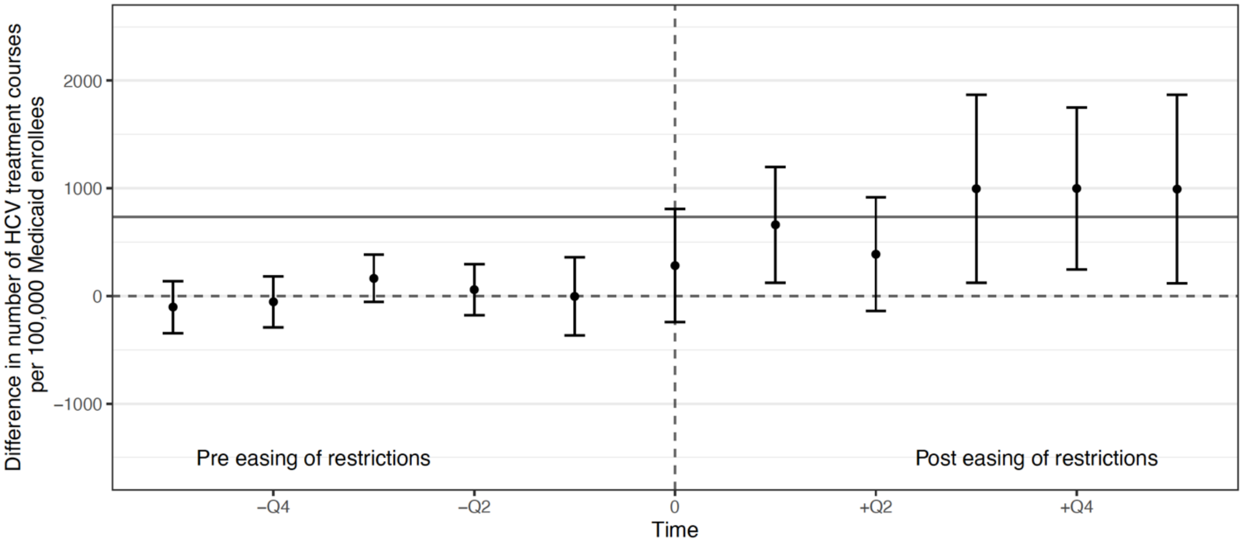

eFigure 2B. Restricted outcome window (+/- 4 quarters).

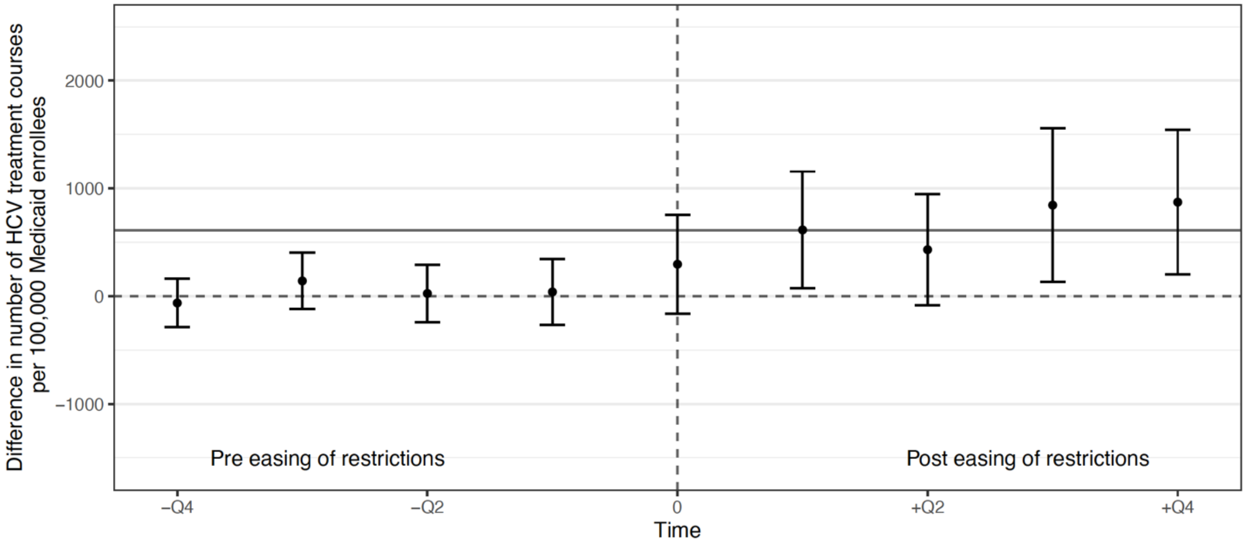

© 2024 Davey S et al. *JAMA Health Forum.*

eFigure 2C. Predominantly fee-for-service states

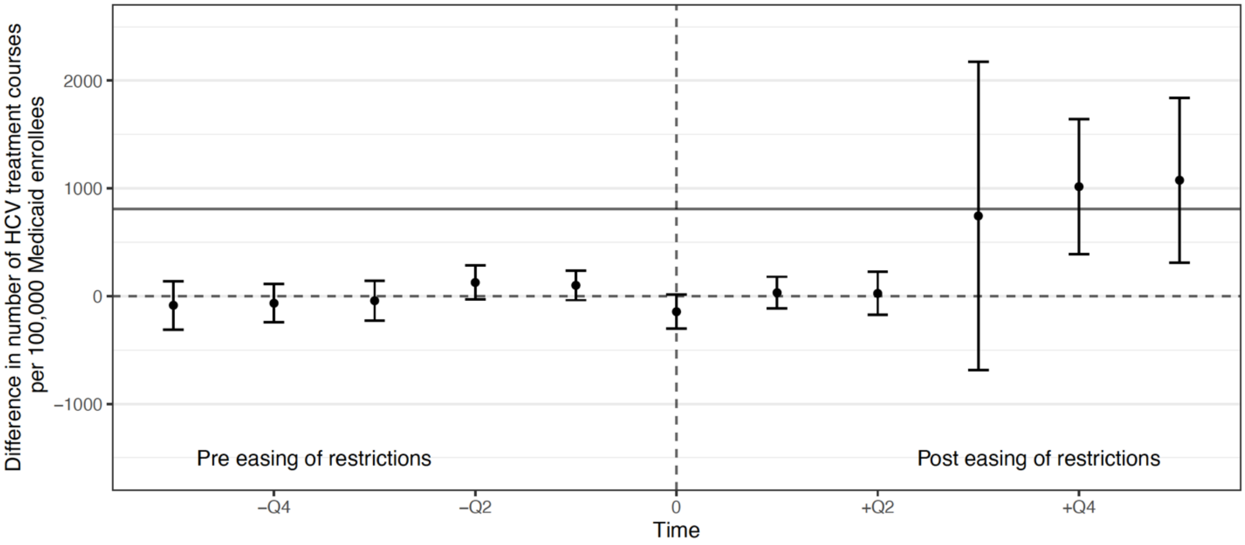

eFigure 2D. Predominantly managed care organization states

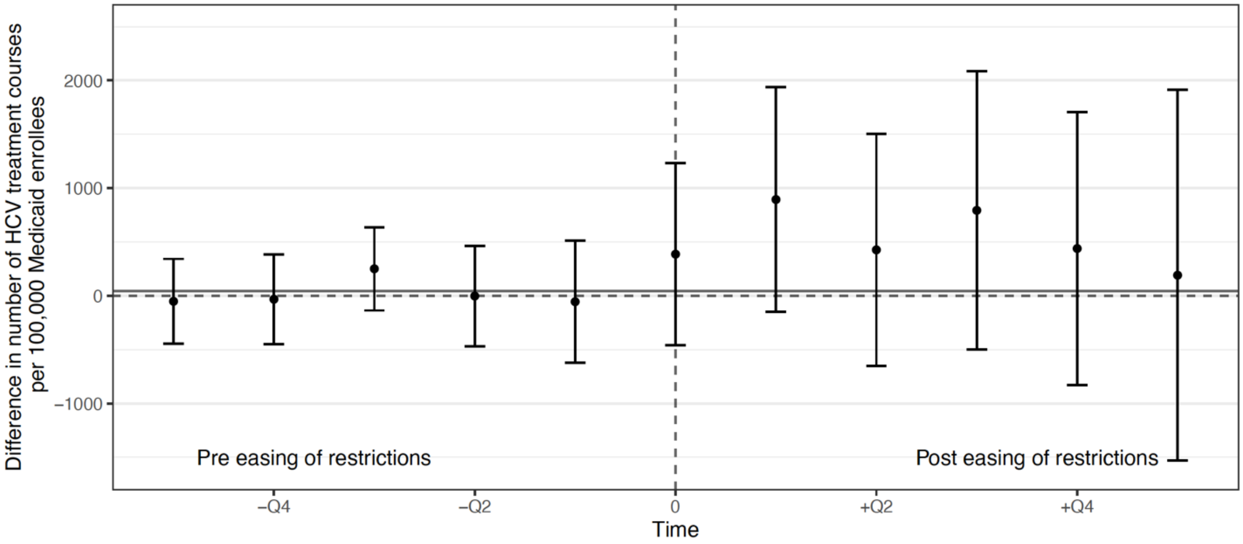

eFigure 2E. Low HCV prevalence states

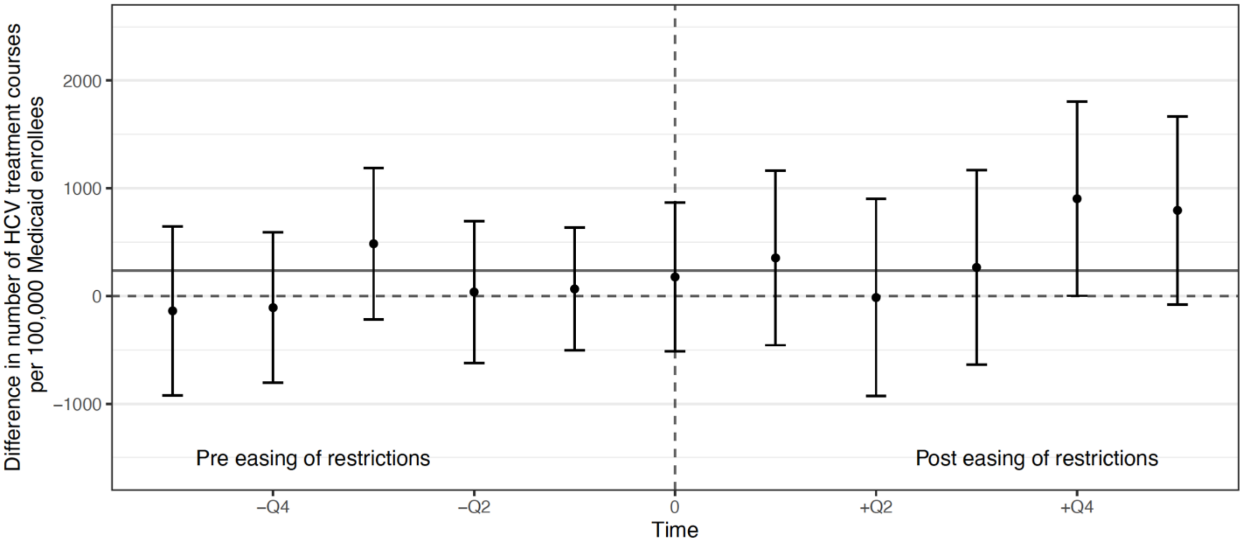

eFigure 2F. Medium HCV prevalence states

© 2024 Davey S et al. *JAMA Health Forum.*

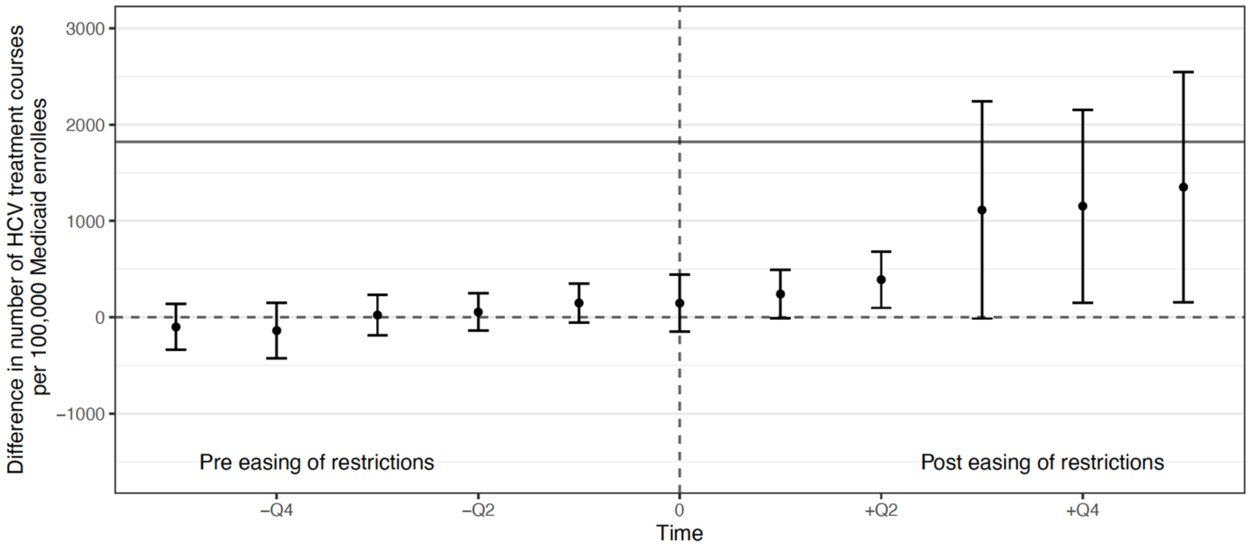

eFigure 2G. High HCV prevalence states

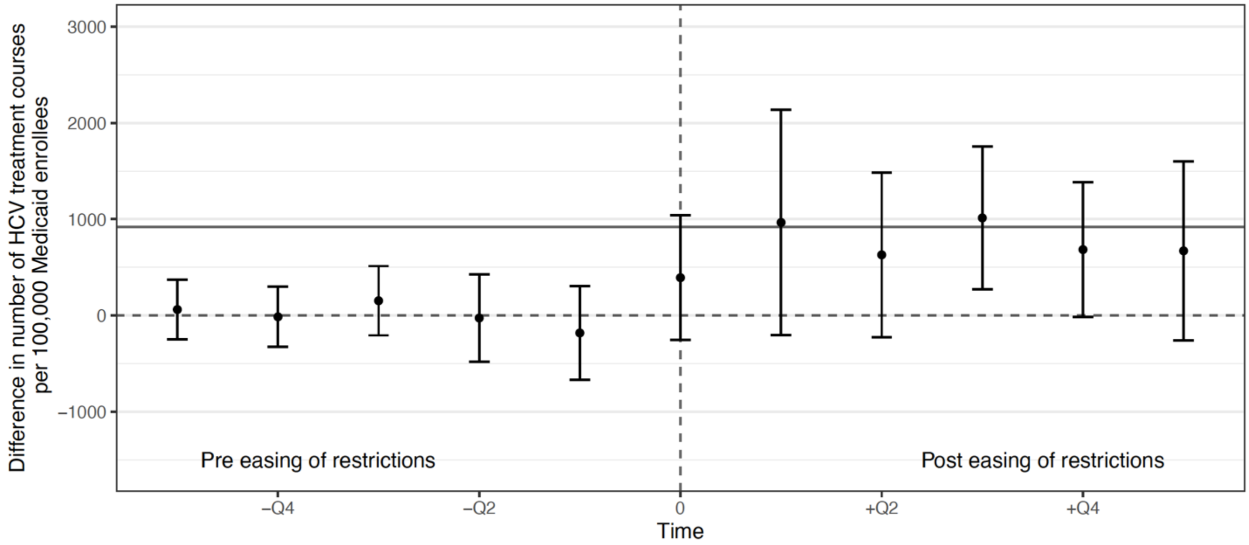

eFigure 2H. States that eased restrictions in 2017 Q2 or earlier

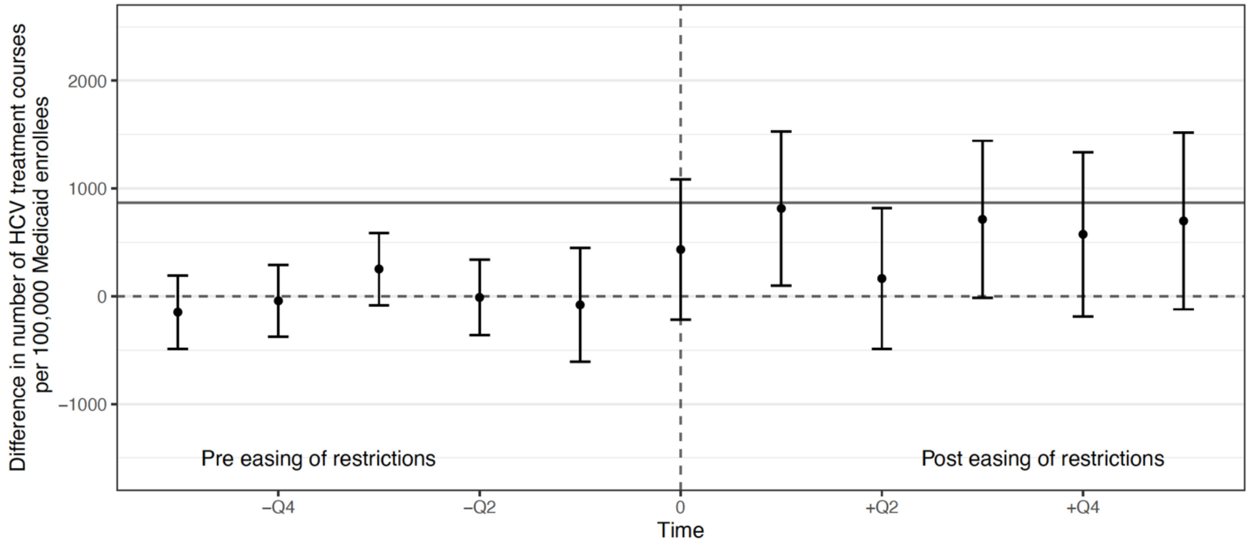

eFigure 2I. States that eased restrictions in 2017 Q3 or later

© 2024 Davey S et al. *JAMA Health Forum.*

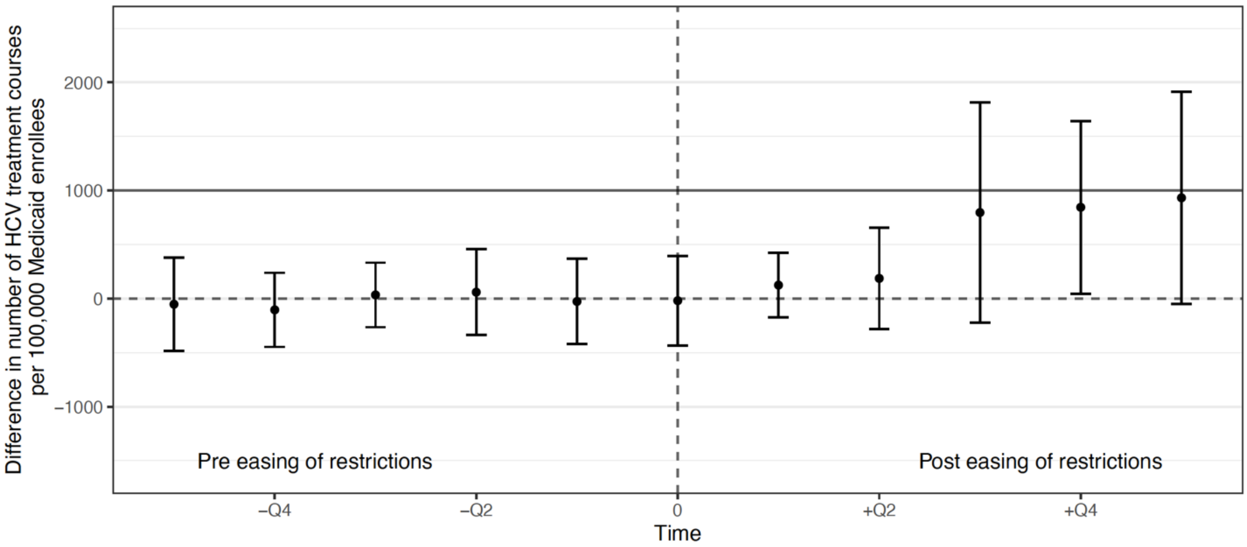

eFigure 2J. States that eased sobriety restrictions

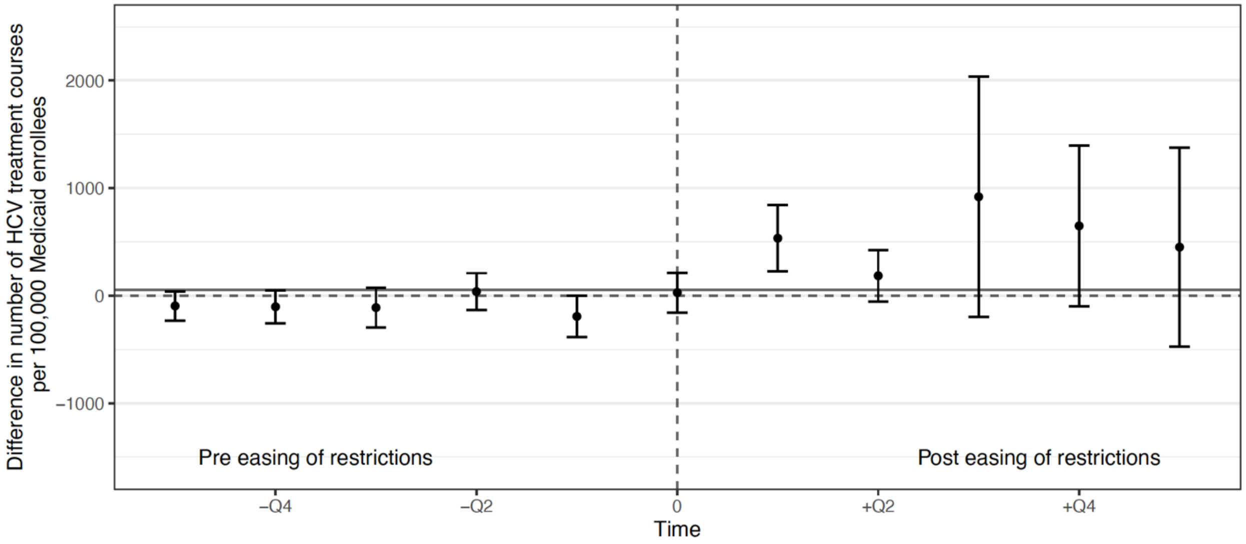

eFigure 2K. States that eased disease severity restrictions

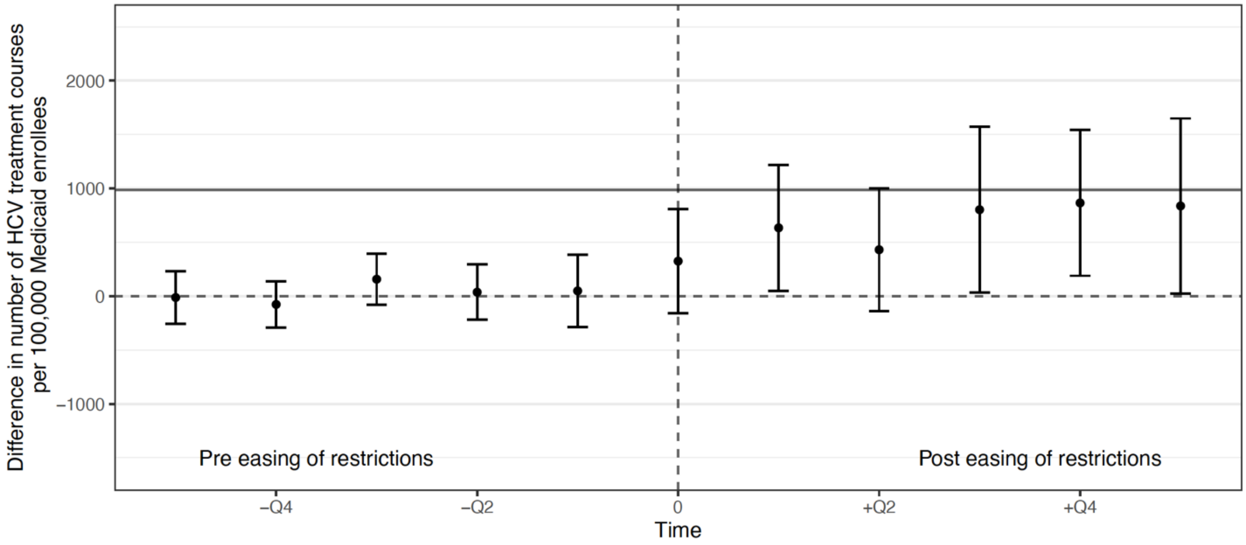

© 2024 Davey S et al. *JAMA Health Forum.*

eFigure 2L. States that eased prescriber restrictions

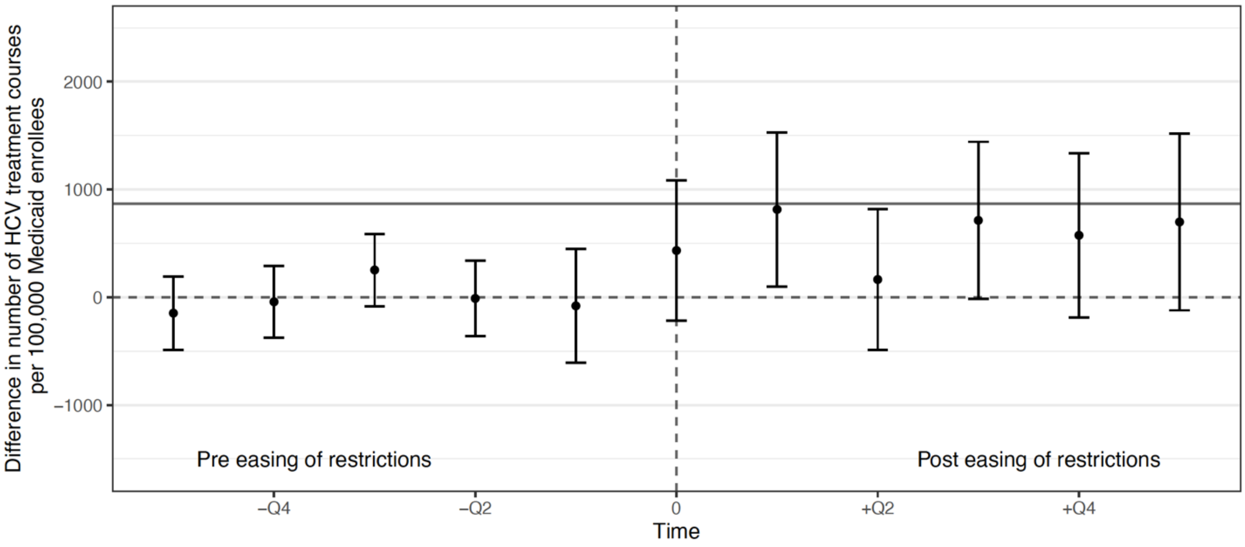

© 2024 Davey S et al. *JAMA Health Forum.*
