# Supplementary material for: Changes in Use of Hepatitis C Direct-Acting Antivirals After Access Restrictions Were Eased by State Medicaid Programs
Source: JAMA Health Forum. 2024 Apr 5;5(4):e240302. doi: 10.1001/jamahealthforum.2024.0302 (PMC10998155; doi:10.1001/jamahealthforum.2024.0302)
Supplement: Supplement 2. — Data sharing statement [file jamahealthforum-e240302-s002.pdf]

Data Sharing Statement

Davey. Changes in Use of Hepatitis C Direct-Acting Antivirals After Access Restrictions Were Eased by State Medicaid Programs. *JAMA Health Forum*. Published April 05, 2024. doi:10.1001/jamahealthforum.2024.0302

Data
Data available: No

Additional Information
Explanation for why data not available: Medicaid State Drug Utilization Data are Publicly available. Data about state restrictions are either available in the supplement or via https://stateofhepc.org/
